# Evaluation of roost culling as a management strategy for reducing invasive rose-ringed parakeet (*Psittacula krameri*) populations

**DOI:** 10.1007/s10530-022-02984-3

**Published:** 2023-01-21

**Authors:** C. Jane Anderson, Leonard A. Brennan, William P. Bukoski, Steven C. Hess, Clayton D. Hilton, Aaron B. Shiels, Shane R. Siers, Bryan M. Kluever, Page E. Klug

**Affiliations:** 1 Caesar Kleberg Wildlife Research Institute, Texas A&M University–Kingsville, Kingsville, TX USA; 2 United States Department of Agriculture, Animal and Plant Health Inspection Service, Wildlife Services, Lihue, Hawai‘i USA; 3 United States Department of Agriculture, Animal and Plant Health Inspection Service, Wildlife Services National Wildlife Research Center, Hawai‘i Field Station, Hilo, Hawai‘i USA; 4 United States Department of Agriculture, Animal and Plant Health Inspection Service, Wildlife Services National Wildlife Research Center, Ft. Collins, CO USA; 5 United States Department of Agriculture, Animal and Plant Health Inspection Service, Wildlife Services National Wildlife Research Center, Barrigada, Guam USA; 6 United States Department of Agriculture, Animal and Plant Health Inspection Service, Wildlife Services National Wildlife Research Center, Florida Field Station, Gainesville, FL USA; 7 United States Department of Agriculture, Animal and Plant Health Inspection Service, Wildlife Services National Wildlife Research Center, North Dakota State University, Department of Biological Sciences, Fargo, ND USA

**Keywords:** Avian, COVID-19, Hawai‘i, Management, *Psittacula krameri*, Rose-ringed parakeet

## Abstract

Rose-ringed parakeets (*Psittacula krameri*) are one of the most widespread invasive avian species worldwide. This species was introduced to the island of Kaua‘i, Hawai‘i, USA, in the 1960s. The rapidly increasing population has caused substantial economic losses in the agricultural and tourism industries. We evaluated the efficacy of a roost culling program conducted by an independent contractor from March 2020 to March 2021. We estimated island-wide minimum abundance was 10,512 parakeets in January 2020 and 7,372 in April 2021. Over 30 nights of culling at four roost sites, approximately 6,030 parakeets were removed via air rifles with 4,415 (73%) confirmed via carcasses retrieval. An estimated average of 45 parakeets were removed per hour of shooter effort. The proportion of adult females removed in 2020 was 1.9 × greater when culled outside of the estimated nesting season. Of the four roosts where culling occurred, the parakeets fully abandoned three and partially abandoned one site. Of the three fully abandoned roosts, an estimated average of 29.6% of birds were culled prior to roost abandonment. The roost culling effort was conducted during the COVID-19 pandemic, when tourist numbers and foot traffic were greatly reduced. It is unknown how public perception of roost culling in public areas may impact future efforts. Findings suggest roost culling can be utilized for management of nonnative rose-ringed parakeet populations when roost size is small enough and staff size large enough to cull entire roosts in no greater than two consecutive nights (e.g., if two shooters are available for three hours per night, roost culling should only be attempted on a roost with ≤ 540 rose-ringed parakeets).

**Supplementary Information:**

The online version contains supplementary material available at 10.1007/s10530-022-02984-3.

## Introduction

Rose-ringed parakeets (*Psittacula krameri*) are one of most widespread and detrimental invasive avian species (Jackson 2021). The popularity of this species in the pet trade has led to introductions worldwide (Menchetti et al. 2016). Escape from the pet trade, combined with this species’ ability to survive in human-modified habitats and tolerance of a wide range of environmental conditions, has led to established populations of rose-ringed parakeets on every continent except Antarctica (Menchetti et al. 2016; ISSC 2021). Throughout much of its introduced range, rose-ringed parakeet populations are increasing. Balmer et al. (2013) estimated the global rose-ringed parakeet breeding range increased over 440-fold from the late 1960s to mid-2010s, making it one of the most rapidly spreading avian species worldwide.

Introduced populations of rose-ringed parakeets have led to negative impacts on native natural resources, economies, and human health and safety. Ecological impacts of invasive rose-ringed parakeets include potential habitat alteration, invasive seed spread, resource competition, and injury and death of native wildlife from the parakeets’ highly antagonistic behavior (Shiels and Kalodimos 2019). In Australia, invasive rose-ringed parakeets have been documented stripping tree bark, resulting in tree death (Fletcher and Askew 2007). Shiels et al. (2018) determined 97% of invasive rose-ringed parakeets evaluated in Hawai‘i, USA, had consumed yellow guava (*Psidium guajava*), potentially leading to seed dispersal of this invasive plant. In Europe, native birds responded to rose-ringed parakeet presence by reducing feeding rates and increasing vigilance (Peck et al. 2014). Perhaps the greatest impact on native species stems from rose-ringed parakeets’ nesting behavior. Rose-ringed parakeets are secondary cavity nesters, utilizing cavities that are naturally occurring or created by other species. In Israel, cavity enlargement by rose-ringed parakeets appears to facilitate breeding success of invasive common mynas (*Acridotheres tristis*; Orchan et al. 2012). In Spain, invasive rose-ringed parakeets maim and kill imperiled greater noctules (*Nyctalus lasiopterus*) to claim cavities, which has led to reduced bat abundance (Hernandez-Brito et al. 2018).

Beyond ecological impacts, invasive populations of rose-ringed parakeets pose hazards to human health and safety. Excessive droppings at roost sites potentially expose humans to zoonotic pathogens such as psittacosis and avian flu (Klug et al. 2019). Furthermore, aircraft have struck rose-ringed parakeets at London’s Heathrow Airport on three documented occasions, which posed a threat to human safety and cost > £20,000 GBP each (Fletcher and Askew 2007). They are also noted agricultural pests in their native and introduced ranges, as they frequently depredate fruit, grain, and seed crops (Khan and Ahmad 1983, Khan et al. 2011; Klug et al. 2019). Rose-ringed parakeets congregate nightly in roosts, which are often in urban, peri-urban, or agricultural areas.

Given the rapidly increasing distribution of rose-ringed parakeets and their pervasive negative impacts, natural resource managers throughout the world are seeking effective population reduction strategies. In parts of their native range, rose-ringed parakeet populations are thought to have been reduced by capture for the pet trade (Menchetti et al. 2016). To date there have been two documented eradications of invasive rose-ringed parakeet populations, both on small islands. A culling program on Mahe Island (157 km^2^) in the Seychelles included the removal of 548 rose-ringed parakeets. The majority of these individuals were removed via shotgun culling along flight lines, with remaining individuals located via a public bounty program (Bunbury et al. 2019). The second documented eradication occurred on La Palma Island Biosphere Reserve (728 km^2^) in the Canary Islands, where 175 individuals were removed over three years. Most of the birds were removed via live trapping, with the final 34 individuals removed via shooting with air rifles (Saavedra and Medina 2020).

Rose-ringed parakeets were introduced to the island of Kaua ‘i, Hawai ‘i, USA, in the 1960s. The population remained relatively low for the following four decades, demonstrating a lag period that is common among introduced parrot populations (Aagard and Lockwood 2014; Menchetti and Mori 2014). The population was estimated to include a minimum of 500 individuals in the year 2000 (Pyle and Pyle 2017). Isolated attempts to reduce crop damage through lethal shooting began in 2005, predominately through use of shotguns at corn (*Zea mays*) fields in the southern part of the island (W. Bukoski, unpublished data). From 2005 to 2019, over 9,000 rose-ringed parakeets were culled by the USDA-APHIS Wildlife Services and private wildlife control operators (Anderson et al. 2021). During this time, there was not a coordinated island-wide control effort; rather, culling was contracted by individual hybrid seed farms. Both the USDA-APHIS Wildlife Services and private wildlife control operators required state-issued depredation permits to cull rose-ringed parakeets. Despite the large removal of rose-ringed parakeets during this time, the population demonstrated marked growth, with abundance estimates of 2,000 individuals in 2011 (Gaudioso et al. 2012) and 6,800 in 2018 (Shiels and Kalodimos 2019). Because Kaua‘i is too remote for unassisted immigration between islands, this increase in parakeet abundance represents growth of the island’s resident population.

Rose-ringed parakeets on Kaua‘i are crop predators of corn, lychee (*Litchi chinensis*), mango (*Mangifera indica*), papaya (*Carica papaya*), longan (*Dimocarpus longan*), and citrus crops, among others (Klug et al. 2019; State of Hawaii Senate Bill No. 772). While no formal economic assessment has been conducted, one hybrid seed production company estimated losses at over US$1 million for commercial hybrid seed corn crops (J. Young, Kani Wildlife Control, *pers. comm.*). The other pronounced impact of rose-ringed parakeets on Kaua‘i results from their roosts, which occur predominantly in urban or peri-urban areas. Like other invaded habitats (Mori et al. 2020), the nightly congregations lead to complaints about inordinate noise, and the excessive droppings lead to property damage (Bernardi et al. 2009; Horman et al. 2017; Spennemann et al. 2017; Klug et al. 2019). Multiple residents and property managers have had to remove or substantially trim palm trees to reduce roosting and associated damage from the parakeets. Commercial property managers on Kaua‘i report that cleaning the droppings requires hours of water use each morning, which in turn leads to mold growth (anonymous commercial property manager, *pers. comm.*). Further, the parakeet droppings appear to attract rodents, potentially as a result of seeds within the droppings (anonymous commercial property manager, *pers. comm.*).

To address the negative impacts of rose-ringed parakeets on Kaua‘i, the State of Hawaii passed House Bill No. 2081 and Senate Bill No. 772; these bills provided funding to evaluate potential control methods and develop an island-wide management plan, funding our analyses described here. Concurrently, a new management effort was implemented in 2020 to reduce the rose-ringed parakeet population on Kaua‘i by culling birds at their roosts; this effort was funded by the County of Kaua‘i and conducted by an independent wildlife control company. Because the rose-ringed parakeet population had grown despite shotgun culling at foraging areas, roost culling was trialed as an alternative. Prior to this effort, roost culling had been generally avoided on Kaua‘i for ecological and social purposes. Primarily, there was concern culling would lead to roost abandonment. This would be problematic because roosts are the primary method of monitoring this species’ abundance. Further, because the roosts were in developed areas, there was concern roost abandonment would lead to new roosts in natural areas. Native songbird species on Kaua‘i are largely restricted to central portions of the island, and expansion of rose-ringed parakeets into this forest interior could be detrimental. Second, two of the three known roosts were adjacent to tourist resorts, and there was concern of how the public would perceive culling efforts. In this regard, reduced tourist activity due to the COVID-19 pandemic provided a unique and optimal timeframe to trial roost culling as a rose-ringed parakeet population management strategy. Only ~ 331,000 tourists traveled to Kaua‘i in 2020, compared to 1.37 million in 2019 (Hawai‘i Tourism Authority 2021). Further, Kaua‘i implemented a 21:00–05:00 curfew from March 20 to May 6, 2020, as a COVID-19 safety precaution (County of Kaua‘i 2020). The company conducting the parakeet culling was granted an exemption from the curfew. The reduced human activity allowed nighttime roost culling to be conducted in public areas with relatively few public observers.

To evaluate the efficacy of the roost culling effort and contribute to the larger island-wide management research, we established a partnership with the company that conducted the roost culling. The company provided us with harvest data and parakeet carcasses. We evaluated overall impact of roost culling on estimated island-wide minimum rose-ringed parakeet abundance. We further evaluated take per hour of shooter effort, estimated the age and sex ratio of animals removed, and monitored whether the parakeets abandoned their roosts in response to culling efforts. Findings can be used to inform implementation and best practices of roost culling for management of nonnative rose-ringed parakeet populations.

## Methods

### Study species

Rose-ringed parakeets are considered a medium-size parrot, with adult body mass ranging from 131 to 180 g (Butler 2003; Mentil et al. 2019). Adults measure around 40 cm in length, about half of which is their long tail. The native range of the four subspecies includes two geographically disjoint regions, with subspecies *P. k. krameri* and *P. k. parvirostris* native to equatorial Africa, and subspecies *P. k. borealis* and *P. k. manillensis* native to the Indian subcontinent (Cardador et al. 2016; Menchetti et al. 2016). Rose-ringed parakeets are one of the few parrot species successfully adapted to living in human-modified environments including urban parks, cultivated areas, and woodlands (Menchetti et al. 2016). Rose-ringed parakeets are sexually dimorphic; adult males are distinguished by black and rose-colored neck rings that develop around three years of age. Although some efforts have been made to distinguish juveniles from adult females by size and plumage (Butler and Gosler 2004; Mentil et al. 2019), they are largely monomorphic (Senar et al. 2019). Nesting season of rose-ringed parakeets on Kaua‘i has not been documented. However, nesting season of rose-ringed parakeets on the neighboring island O‘ahu has been observed as late January to mid-April (Shiels and Kalodimos 2019).

### Study area

**Fig. 1:**
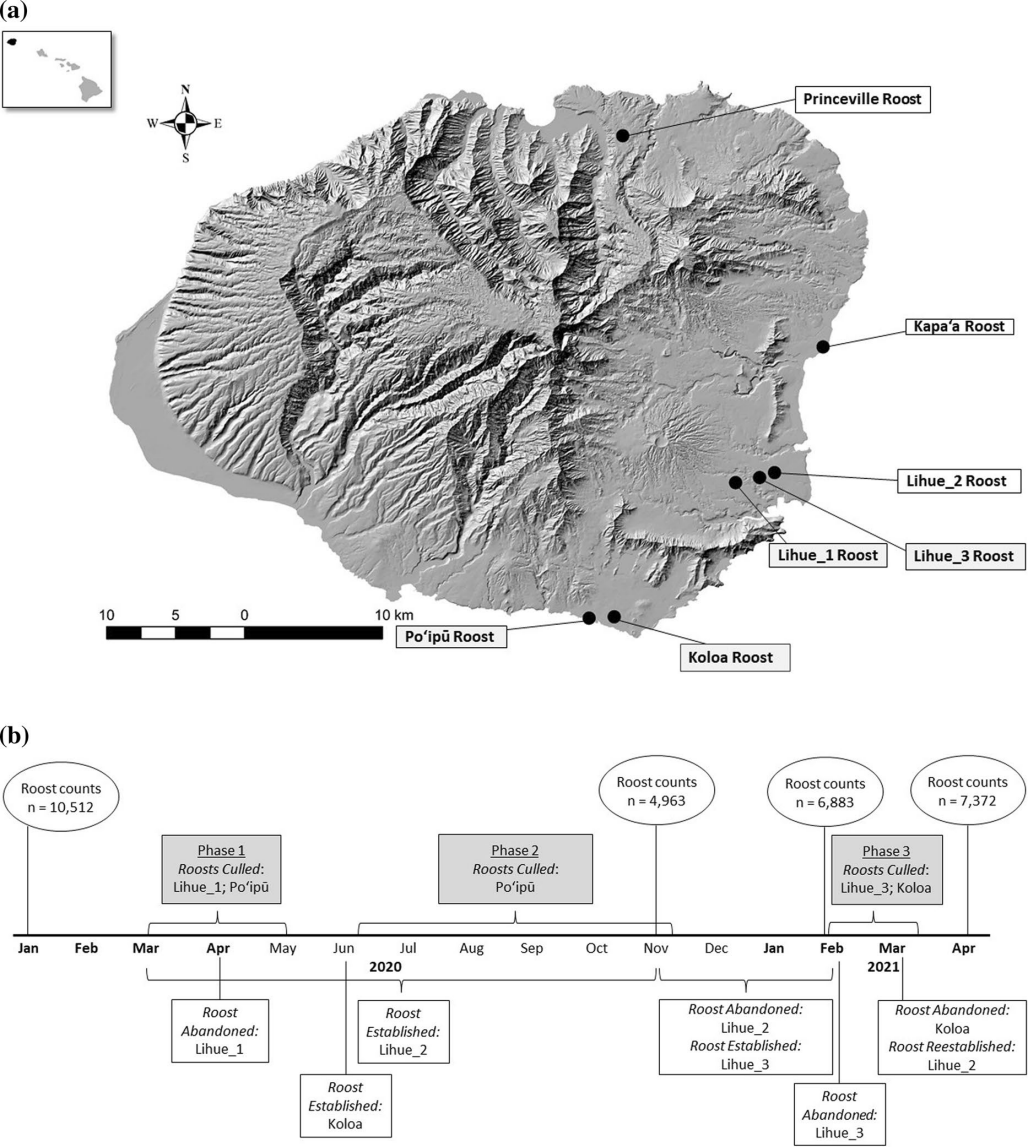
**a** Rose-ringed parakeet (*Psittacula krameri*) roosts observed on Kaua‘i, Hawai‘i, in 2020 and 2021, and **b** timeline of roost counts (ellipses), culling phases (grey shading), and observed roost establishment and abandonment dates (white shading). Brackets are used to indicate roost abandonment or establishment when the exact date was not known. Months in bold (January–April) represent estimated reproductive season. Reported roost counts represent estimated island-wide minimum abundance (Table 1)

The Hawaiian Island archipelago is among the most remote in the world. It consists of eight major islands and dozens of smaller islets. Kaua‘i is the northernmost and fourth largest of the major islands (Fig. 1). It has an area of 1,430 km^2^ and ranges in elevation from sea level to 1,598 m. Kaua‘i has a tropical climate, with average daily temperatures of 26 °C in February and 29 °C in August. January–March is regarded as the wet season, although precipitation is high year-round; mean annual rainfall ranged from 440 mm in the lowlands to nearly 10,000 mm at the highest peak (Giambelluca et al. 2013).

### Study design

We estimated the island-wide minimum abundance of rose-ringed parakeets on Kaua‘i prior to, during, and post roost culling by conducting roost counts – the method commonly used to estimate rose-ringed parakeet population size (Butler 2003; Pithon and Dytham 1999; Shiels and Kalodimos 2019; Fig. 1) – on all known roosts on the island (Table 1; Fig. 1). Roosts were identified by public reports and surveys conducted by our study team prior to the culling effort. Roost counts were conducted by counting rose-ringed parakeets as they departed the roost at sunrise; we did not count birds when they returned to the roosts at sunset, as we found they returned from too many directions and moved between trees too rapidly to reliably count them. When flocks were too large to count by individual, parakeets were counted by increments of five or ten individuals (Shiels and Kalodimos 2019) using methods described by Meanley (1965) and Arbib (1972). Each roost count was conducted two to four times within a two-week period, and counts were averaged to represent the respective estimate (Table 1; Fig. 1). This technique should be considered a conservative population estimate, or minimum number of known individuals, as it is possible there were unknown roosts during any given time; thus, we refer to cumulative estimates from roost counts as island-wide minimum abundance.

**Table 1:** Estimates of island-wide minimum abundance of rose-ringed parakeets (*Psittacula krameri*) on Kauai, Hawai‘i, USA, estimated via roost counts. Roost counts were conducted two to four times at each site within a two-week period; reported estimates represent averages from the respective time period. Periods indicate the roost was not occupied during the respective roost count

Roost	January 2020	November 2020	February 2021	April 2021
Kapa‘a	1,501	1,911	1,657	1,358
Lihue_1	2,559	.	.	.
Lihue_2	.	880	.	1,317
Lihue_3	.	.	1,031*	.
Poʻipū	6,452	1,325	3,043	4,650
Koloa	.	8,47	1,152	.
Princeville	.	.	.	47
Total	10,512	4,963	6,883	7,372

*This estimate was conducted prior to the February 2021 roost culling at the Lihue_3 roost; an estimated 725 birds were located at Lihue_2 after roost culling.

Prior to roost culling we estimated island-wide minimum abundance in January 2020, at which time there were three known roosts: Poʻipū, Lihue_1, and Kapa‘a (Fig. 1). The wildlife control company paused roost culling from November 2020 to February 2021. We estimated island-wide minimum abundance twice during this period, once immediately after culling cessation in November 2020 and once immediately before culling was resumed in February 2021. In November 2020 there were four known roosts: Poʻipū, Lihue_2, Koloa, and Kapa‘a. In February 2021 there were four known roosts: Poʻipū, Koloa, Lihue_3, and Kapa‘a. In April 2021, we estimated minimum island-wide abundance after the completion of culling, at which time there were four known roosts: Poʻipū, Lihue_2, Kapa‘a, and Princeville (Fig. 1).

Thirty nights of roost culling were conducted from March 2020 to March 2021 divided among three phases (Fig. 1). Phases were differentiated by whether they included the estimated nesting season (approximately January–mid April), number of roosts culled, and culling frequency (see below). For each night of culling, we documented the estimated number of parakeets lethally removed from the roost, total number of carcasses retrieved, number of shooters, and number of hours culling was conducted. Not all carcasses were retrieved, as some were caught in the vegetation and did not fall into the ground, others were immediately scavenged by feral cats, and some birds flew away with injuries and could not be located (J. Young, Kani Wildlife Control, *pers. comm.*). In our original study design, we intended for a member of our team to accompany all culling activities; however, travel restrictions due to the COVID-19 pandemic prohibited us from attending all but three nights of culling. All data – estimates of parakeets removed nightly, number of carcasses retrieved, number of shooters, and number of hours worked - were provided by the private company that implemented the culling effort, Kani Wildlife Control (Kekaha, HI, USA). Culling was conducted by one or two shooters each night using sound-suppressed 22-caliber air rifles with telescopic sights. Each shooter had a partner using a white spotlight to locate parakeets. We calculated the total shooter hours for each night as the number of hours worked per shooter (e.g., two shooters working for three hours = six shooter hours). We calculated the estimated number of birds culled and total number of carcasses retrieved per shooter hour and the means (± standard deviations) for each phase.

#### Roosts subjected to culling:

The roosts of rose-ringed parakeets on Kauaʻi were in urban to peri-urban habitats. We measured the approximate size of each roost by documenting the coordinates of the two furthest points of the roost and measuring the distance between them using ArcMap (version 10.6.1, ESRI, Redlands, CA, USA). The Poʻipū roost included > 100 ornamental trees over approximately 1 km; trees used for roosting by the rose-ringed parakeets included royal palms (*Roystonea regia*) and king palms (*Archontophoenis alexandrea*). Within this area were four tourist resorts, the edge of a golf course, a public park, and > 50 private residential properties. The Lihue_1 roost included 46 royal palms and two coconut palms (*Cocos nucifera*) concentrated in the parking lot of a shopping center; total length of the roost was ~ 190 m. The Lihue_3 roost was not occupied at the beginning of the study; it had historically been a roost site less than six months prior to the study (CJ Anderson, *pers. obs.*) and was repopulated by the parakeets between November 2020 to February 2021. This roost was spatially the smallest of all evaluated, with a total length around 60 m. In this site, the parakeets were using ~ 20 royal palms in a lawn surrounding a convention center. The Koloa roost was not occupied at the beginning of the study but was also historically a roost site, with documented use in 2011 (Gaudioso et al. 2012). The parakeets reestablished the Koloa roost around June 2020 (WP Bukoski, *pers. obs.*) in a row of ~ 50 palm trees along a road between a residential street and a golf course. The length of the roost was approximately 237 m (Fig. 1).

#### Phase 1: March–May 2020

Twenty-one nights of culling were conducted from March 27 to May 29, 2020, between two roosts, Lihue_1 and Poʻipū (Fig. 1). This Phase was conducted during the predicted nesting season of rose-ringed parakeets on Kaua‘i. No culling was conducted at the third known roost at the time, the Kapa‘a roost. The Lihue_1 roost was markedly smaller in estimated number of birds occupying the roost and spatial length than the Poʻipū roost (see above). There were four nights of culling (10.68 shooter hours) at the Lihue_1 roost, (March 27 – April 17, 2020), implemented at a rate of once per week. There were 17 nights of culling (64.67 shooter hours) at the Poʻipū roost (April 1 – May 29). This included five nights of culling (15.13 shooter hours) in April 2020 implemented at a rate of once per week and 12 nights of culling (49.53 shooter hours reported for nine nights; data were not provided for three) in May 2020 implemented at an average rate of every 2.5 days (min = 1, max = 6).

We obtained 2,993 carcasses from the parakeets culled in Phase 1. We documented the total number of adult males, as noted by their black and rose-colored neck collars. Birds lacking neck rings were initially classified as unknown age and sex. From the sample of unknown birds, we conducted 601 necropsies to determine age and sex in three classes: sexually mature females, immature females, and immature males; we randomly selected carcasses to necropsy by culling date. We determined sex by presence of testes or ovaries. Immature males were classified as those with testes but lacking neck rings. We determined female age by follicular development; females in which all follicles were undeveloped were designated as sexually immature, and those with active follicles (visually identified by size) were designated as sexually mature (J. Heatley, Texas A&M University, *pers. comm.*; Fig. S1 in Supplementary Information). Of the 601 carcasses, 16 had too much internal damage to accurately assess age and sex, leading to a usable sample of 585 carcasses (32% from the Lihue roost; 68% from the Poʻipū roost). Our sample size (n = 585) was sufficient to estimate the proportion of all culled birds by age and sex (95% confidence level, 5% margin of error; Cochran 1977). We therefore used the proportion of each age and sex class among necropsied birds to estimate the proportion of the respective age and sex class in the total sample (n = 2,993). Using these findings, we estimated the proportion and 95% confidence interval of each age and sex class of parakeets removed during the entire study period.

#### Phase 2: June–November 2020

Phase 2 was a continuation of culling efforts at the Poʻipū roost (Fig. 1). We evaluated this period separately as it did not include the predicted nesting season and differed in culling frequency. There were six nights of culling between June 5 and November 12, 2020 (27 shooter hours). Frequency of culling effort was less consistent during this time as compared to Phase 1, with an average of 32 nights between culling efforts (min = 1, max = 85). The largest lag between culling events was from August to November 2020. We were unable to evaluate age and sex of carcasses retrieved during this time due to COVID-19 travel restrictions.

#### Phase 3: February & March 2021

Two nights of culling were implemented at the Lihue_3 roost on consecutive nights, 23–24 February 2021 (17.5 shooter hours; Fig. 1). To evaluate the impact of successive disturbance on roost abandonment, we conducted roosts counts at the Lihue_3 roost on February 22, 24, and 26, 2021. For all carcasses collected at the Lihue_3 roost (n = 567), we recorded the number of adult males and number of unknown age and sex birds. From the first of the two nights of culling, we necropsied 52 of the unknown birds to estimate the age and sex ratio of the parakeets removed. Following the protocol of Phase 1, we assumed the age and sex ratio of carcasses we did not necropsy (n = 165) were the same as those we did.

The final night of roost culling was conducted at the Koloa roost (Fig. 1) in March 2021 (9.32 shooter hours). The company conducting the culling reported the estimated number of parakeets removed (n = 214) and reported total number of carcasses retrieved (n = 165) but did not obtain carcasses to evaluate age and sex.

#### Comparison of age/sex culled by season

We compared age and sex ratio of rose-ringed parakeets culled by season. During the nesting season, nesting female rose-ringed parakeets remain on their nests rather than returning to the roost at night—beginning when the first egg is laid (Braun and Wink 2013)—and are therefore unlikely to be at the roost during culling. We therefore evaluated whether age and sex ratio differed between birds removed in March and April 2020 (n = 1,140) with those in May 2020 (n = 1,853) during Phase 1. While February is within the estimated nesting season, post-hoc observation indicated a higher proportion of adult females were culled in February 2021 (n = 402). We performed a multinomial logistic regression using the *nnet* package in R (version 4.0.5) with age/sex as the response variable (mature female, mature male, immature female, and immature male) and culling season as the independent variable (March/April 2020, May 2020, February 2021) to evaluate whether age and sex ratio of parakeets culled varied significantly by season (R code provided in Supplementary Information).

## Results

We estimated an island-wide minimum abundance of 10,512 rose-ringed parakeets in January 2020 (prior to roost culling) among the three known roosts (Table 1). An estimated 6,030 parakeets were removed via roost culling with 4,415 carcasses retrieved from March 2020 – March 2021. Among all culling efforts, there was an estimated mean of 44.7 (SD = 28.4) parakeets removed per shooter hour. An average of 36.7 (SD = 22.9) carcasses were retrieved per shooter hour (Table 2). Island-wide minimum abundance estimates were 4,963 individuals in November 2020, 6,883 in February 2021, and 7,372 in April 2021 (Table 1). The number, location, and estimated abundance of roosts changed throughout the study.

**Table 2:** Estimated number of rose-ringed parakeets (*Psittacula krameri*) culled and carcasses retrieved from roost culling efforts on Kaua‘i, Hawai‘i, USA

	Total	Phase 1	Phase 2	Phase 3	
		March – April 2020 (Lihue_1 & Poʻipū)	May 2020 (Poʻipū)	June – November 2020 (Poʻipū)	February—March 2021 (Lihue_3 & Koloa)
Nights of culling	30	9	12	6	3
Estimated parakeets culled	6,030	1,511	3,415	392	712
Carcasses retrieved	4,415	1,277	2,333	238	567
Mean estimated take per shooter hour (SD)	44.7 (28.4)	53.8 (25.9)	56.25 (23.7)	22.2 (29.6)	28.1 (16.6)
Mean carcasses retrieved per shooter hour (SD)	36.7 (22.9)	49.6 (19.9)	39.4 (17.1)	19.5 (29.1)	22.6 (15.3)

### Phase 1: March–May 2020

Over 21 nights of culling, an estimated 4,926 rose-ringed parakeets were removed from the Lihue_1 and Poʻipū roosts, a confirmed 3,619 carcasses were retrieved, and average take per shooter hour was 55.0 (SD = 23.5). At the Lihue_1 roost, an estimated 561 parakeets were removed (530 carcasses retrieved) over four nights of culling, approximately 22% of the pre-culling roost abundance (n = 2,559; Table 1). During the fourth night of culling, the number of birds removed per shooter hour decreased substantially (13.1) from the average of the first three nights (59.8), as the parakeets had partially abandoned the roost (J. Young, Kani Wildlife Control, *pers. comm.*). After the fourth night of culling, the parakeets fully abandoned the roost.

An estimated 4,365 parakeets were removed from the Poʻipū roost—approximately 68% of the pre-culling roost abundance (6,452; Table 1)—and 3,089 carcasses were retrieved. Average take per shooter hour was 57.0 (SD = 18.6). Over the 17 nights of culling, the parakeets did not fully abandon the roost. While we did not measure the number of birds per tree or per individual property, we noted that the extent and composition of the roost changed. For example, during this period, the parakeets shifted approximately 175 m west onto a different property. In many areas, the parakeets shifted to private properties where culling was not permitted (J. Young, Kani Wildlife Control, *pers. comm.*).

**Fig. 2:**
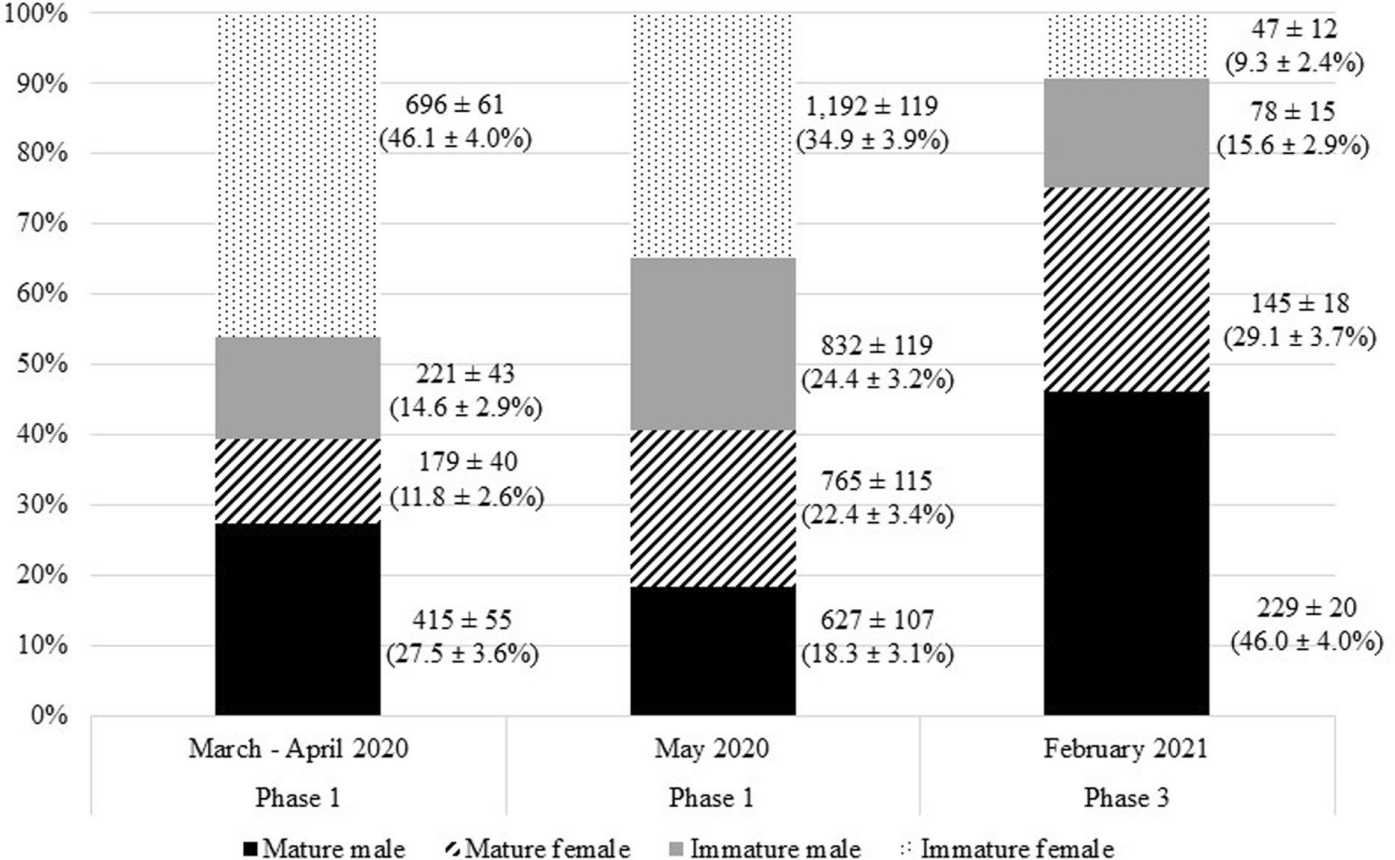
Estimated number and percent (95% CI) of rose-ringed parakeets (*Psittacula krameri*) by age and sex removed via roost culling from March—April 2020 (n = 1,511 from Lihue_1 and Poʻipū roosts), May 2020 (n = 3,415 from Poʻipū roost), and February 2021 (n = 498 from Lihue_3 roost) on Kaua‘i, Hawai‘i, in 2020 and 2021. We identified all mature males by plumage, whereas we identified a sample of mature females, immature females, and immature males via necropsy during Phase 1 (n = 337 from March – April; n = 248 from May) and Phase 3 (n = 52). Carcasses were not evaluated during Phase 2 due to COVID-19 travel restrictions

Of the 2,993 carcasses evaluated, 653 (21.8%) were sexually mature males. Of the 585 carcasses necropsied, 142 were immature males, 123 were mature females, and 320 were immature females (Table 3). Using these findings to estimate the total samples size, we estimated the 4,926 parakeets removed during Phase 1 included 21.8 ± 3.4% mature males, 19.0 ± 3.2% immature males, 16.4 ± 3.0% mature females, and 42.7 ± 4.0% immature females (Fig. 2).

**Table 3:** Observed age and sex of rose-ringed parakeets (*Psittacula krameri*) removed via roost culling on Kaua‘i, Hawai‘i, USA, in March—May 2020 (n = 2,993, Phase 1) and February 2021 (n = 402, Phase 3). We identified all mature males by plumage, whereas we identified a sample (n = 585, Phase 1; n = 52, Phase 3) of mature females, immature females, and immature males via necropsy. Carcasses were not evaluated during Phase 2 due to COVID-19 travel restrictions

	Phase 1	Phase 3	
	March – April 2020	May 2020	February 2021*
Mature males	313	340	185
Unknown age/sex (not necropsied)	490	1,265	165
Immature males	68	74	15
Mature females	55	68	28
Immature females	214	106	9
Total	1,140	1,853	402

*includes only individuals culled at the Lihue_3 roost

### Phase 2: June–November 2020

**Fig. 3:**
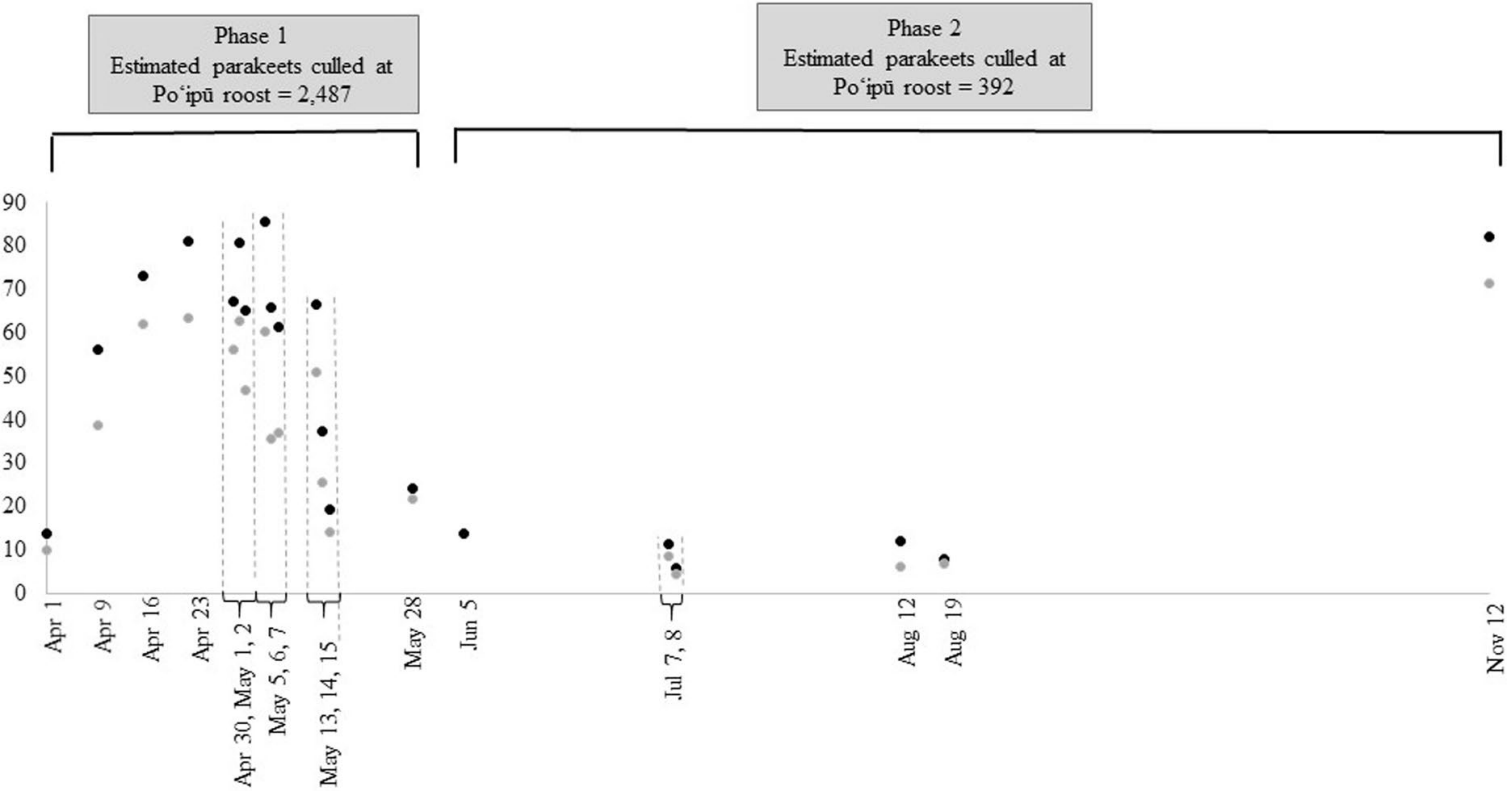
Estimated number of rose-ringed parakeets (*Psittacula krameri*) culled per shooter hour (black dots) and total number of carcasses retrieved per shooter hour (gray dots) at the Poʻipū roost in 2020 (Phases 1 and 2) on Kaua‘i, Hawai‘i, USA. Data were not reported by the independent wildlife control company for three of the 23 nights included in this time period (May 21, 22, and 29)

An estimated 392 rose-ringed parakeets were culled (238 retrieved carcasses) at the Poʻipū roost. The average number of parakeets removed per shooter hour in Phase 2 (22.2 [SD = 29.6]; Table 2) was lower than Phase 1 and ranged from 7.8 to 13.7 in June–August. After an 85-day hiatus in culling, the number of birds removed per shooter hour increased in November to 82.3 (Fig. 3).

We estimated the island-wide minimum abundance was 4,963 individuals among four roosts in November 2020 after the conclusion of Phase 2 roost culling. Given that the Lihue_1 roost was abandoned during roost culling, there were no birds in this location. The Poʻipū roost was greatly reduced in size due to culling and partial roost abandonment. The Kapa‘a roost, where no culling occurred, had increased by approximately 27%. Two roosts had established, Lihue_2 and Koloa; it was unknown when the Lihue_2 roost established, but the Koloa roost was first documented in June 2020 (Fig. 1; Table 1; W. Bukoski, *pers. obs.*). While neither roost was present in January 2020, both were historic roost locations that had previously been abandoned for unknown reasons.

### Phase 3: February & March 2021

Between November 2020 and February 2021, the Lihue_2 roost abandoned for unknown reasons and the Lihue_3 roost became established (Fig. 1). Among the four known roosts in February 2021, we estimated an island-wide minimum abundance of 6,883 rose-ringed parakeets (Poʻipū, Lihue_3, Kapa‘a, and Koloa; Table 1).

We estimated the Lihue_3 roost comprised 1,031 birds prior to culling. On 23 February 2021, an estimated 350 rose-ringed parakeets were culled (298 retrieved carcasses). We estimated 680 parakeets were occupying the Lihue_3 roost on the morning of 24 February; that night, an estimated 148 additional parakeets were culled (103 retrieved carcasses). We counted only 14 parakeets at the roost on the morning of 26 February, indicating the site had been effectively abandoned. Between the two nights of culling, an estimated 48.3% of the roost was culled prior to abandonment.

Of the 402 carcasses retrieved, 185 (46.0%) were mature males. Of the 52 birds of unknown age and sex, we identified 28 mature females, nine immature females, and 15 immature males via necropsy (Table 3). Using these observations, we estimated take from the Lihue_3 roost during this period included 29.1 ± 3.7% sexually mature females, 15.6 ± 2.9% immature males, and 9.3 ± 2.4% immature females (Fig. 2). Qualitatively, we observed that follicles were more developed among females in February 2021 than March–May 2020, including one female with an intact egg.

During the single night of culling at the Koloa roost, an estimated 214 individuals were removed (165 retrieved carcasses). The parakeets did not return the following evening, indicating they abandoned the site after only one night of culling. An estimated 18.6% of the roost was removed prior to abandonment.

### Comparison of age/sex culled by season

The age and sex ratio of rose-ringed parakeets culled varied between the March–April 2020 (n = 1,140), May 2020 (n = 1,853), and February 2021 (n = 498) sampling periods (Table 3; Fig. 2). The likelihood of culling a mature female vs an immature female, immature male, or mature male was significantly higher in February compared with March/April or May (*p* < 0.01; Table 4).

**Table 4:** Multinomial logistic regression predicting proportion of mature females compared with mature males, immature females, and immature males culled via roost culling on Kaua‘i, Hawai‘i, USA, over three time periods: March/April 2020 (n = 1,511), May 2020 (n = 3,416), and February 2021 (n = 499). Because the percentage of adult females was highest in February, this month was used as the reference level

	Coefficient March/April | May	Standard error March/April | May	*P*-value March/April | May
Mature males	0.38 |− 0.66	0.14 | 0.12	< 0.01 | < 0.01
Immature females	2.48 | 1.57	0.19 | 0.17	< 0.01 | < 0.01
Immature males	0.83 | 0.70	0.17 | 0.15	< 0.01 | < 0.01

## Discussion

Roost culling appears to be an efficient mechanism to rapidly remove large numbers of rose-ringed parakeets with relatively little effort. In this study, an estimated > 6,000 parakeets were removed in one year at a cost of around $30,000 US (J. Young, Kani Wildlife Control, *pers. comm.*). Comparatively, the effort to remove < 600 rose-ringed parakeets in the Seychelles cost around US $1 million (Bunbury et al. 2019). However, our findings indicated roost culling is an imperfect population control strategy. The roost abandonment observed in this study as a result of roost culling is arguably the largest drawback of this methodology. In future implementation of roost culling, roost abandonment should be avoided, as it may compromise population monitoring (Bunbury et al. 2019), potentially disturb native species (e.g., by roost reestablishment in forested areas on Kauaʻi), and condition surviving rose-ringed parakeets to avoid future culling efforts.

All roosts subjected to roost culling in this study were either fully or partially abandoned by rose-ringed parakeets. The likelihood and rate of abandonment appeared to be related to roost size, roost land cover, as well as exposure of parakeets to previous roost culling. Two roosts were culled in Phase 1 and therefore included parakeets without prior exposure to roost culling. The Lihue_1 roost was partially abandoned after three nights of weekly roost culling and fully abandoned after four nights. The Poʻipū roost was never fully abandoned; however, this roost was spatially restructured and partially abandoned by the parakeets. The lack of complete abandonment may have been due to the large size of the Poʻipū roost, which allowed shooters to operate in sections of the roosts (e.g., individual properties) rather than disturbing the entire roost simultaneously (J. Young, Kani Wildlife Control, *pers. comm.*). The two roosts culled in Phase 3 (Koloa and Lihue_3) were both established after other roosts had been abandoned in response to roost culling. Thus, it is feasible the birds in these roosts came from the abandoned roosts and therefore had previous exposure to roost culling. Parakeets in these roosts fully abandoned after one and two nights, respectively. These observations suggest surviving rose-ringed parakeets learn to avoid roost culling and will abandon roosts more quickly in subsequent culling efforts. Collectively, these findings indicate roost culling is best used for roosts spatially large enough (≥ 1 km) to cull in sections, or small enough in abundance to cull entirely in no more than two nights. The optimal frequency of roost culling implementation, including whether consecutive nights of culling should be utilized, is difficult to ascertain from this study. Future research should further investigate the impact on culling frequency on roost abandonment.

We found an average of ~ 45 rose-ringed parakeets can be culled per shooter hour via roost culling; managers can use this estimate to determine whether roosts are small enough and staff size is sufficient to successfully remove all birds from the roost prior to abandonment (e.g., if two shooters are available for three hours per night, roost culling should only be attempted on a roost with ≤ 540 rose-ringed parakeets). While the Poʻipū roost was not fully abandoned despite culling throughout Phases 1 and 2, take per shooter hour substantially decreased during Phase 2 (Fig. 3). This finding indicates that parakeet exposure to roost culling or decreased roost size may influence efficacy of this strategy. It is important to note that the shooters in this study were wildlife control professionals with years of experience culling birds; less experienced shooters would likely have a lower take rate.

In implementing roost culling, managers should work to prevent conditioning surviving individuals to avoid culling. On the nights we attended roost culling efforts, we observed parakeets flushing in response to the white spotlights. Use of infrared or thermal spotting technologies would likely be less noticeable to the birds (Klug et al. 2023). The firearms in this effort included sound suppressors, which are ideal both to prevent flushing parakeets and avoid disturbing other species (Klug et al. 2023).

In this study, approximately 30% of rose-ringed parakeet carcasses were not retrieved during nightly culling efforts, raising both ethical and public health concerns. It is unknown how many unretrieved birds died immediately but did not fall to the ground or were consumed by scavengers versus how many experienced sublethal injuries. The roost culling effort on Kauaʻi was conducted by a private company; similar efforts by government agencies or academic institutions would have more stringent requirements of ensuring humane euthanasia. Regardless of whether it is mandated, we recommend managers implementing roost culling work to locate all injured animals and include a secondary euthanasia protocol for injured animals in compliance with the American Veterinary Medical Association Guidelines for the Euthanasia of Animals; these methods include inhaled gases (e.g., CO_2_) or cervical dislocation (Leary et al. 2020). In addition to ensuring all animals are humanely euthanized, future roost culling efforts should continue to safely remove and dispose of all carcasses. Unretrieved carcasses are unsightly, unsanitary, may be objectionable to the public (Klug and Homan 2020), and may subsidize invasive scavengers (e.g., cats [*Felis catus*] and rodents).

The island-wide minimum abundance of rose-ringed parakeets on Kaua‘i decreased by an estimated > 3,000 birds from between January 2020 and April 2021 (Table 1), marking the first documented annual population decrease. This was particularly noteworthy given there were two estimated reproductive seasons during this period (Gaudioso et al. 2012; Shiels and Kalodimos 2019). Roost culling in 2020 resulted in the removal of approximately 5,318 rose-ringed parakeets. In addition to roost culling, approximately 2,500 rose-ringed parakeets were removed on Kaua‘i in 2020 via shotgun culling at corn fields (J. Young, Kani Wildlife Control, *pers. comm*; T. Kaiakapu, Hawaii Division of Forest and Wildlife, *pers. comm*). Demographic parameters such as fecundity, survivorship, and annual growth rate are unknown on Kaua‘i. However, the finding of a minimum abundance of > 7000 individuals in April 2021 despite the removal of > 7,800 individuals in the preceding year (between roost and shotgun culling) indicates the January 2020 abundance estimate in this study was an underestimate of true population size. After the conclusion of this study, an island-wide community science effort was conducted to locate roosts; this effort resulted in the identification of two roosts that were established in 2020 and 2021 according to reports by local residents (Anderson et al. 2021). Continued research to locate rose-ringed parakeet roosts and more precisely estimate population size is merited and will be integral to future management efforts.

The size, location, and observed abandonment of roost sites were critical considerations of this study and should be evaluated in all future roost culling efforts. In comparing the number of individuals per roost before and after culling, we assumed rose-ringed parakeets did not move among roosts during the study period. While it is unknown whether this occurs, evidence suggests that if it does occur, it is not common. We observed the reoccupation of previously abandoned roosts (Lihue_2, Lihue_3, and Koloa) and, in the largest roost, the shifting of the birds to protected properties (Poʻipū). The relatively consistent size of undisturbed roosts over time suggests that rose-ringed parakeets do not frequently move between roosts; however, if this did occur during the study period, it may have influenced our findings. Rose-ringed parakeets will sometimes abandon roosts for unknown reasons in the absence of management (Peck 2013; Pyle and Pyle 2017). Roost abandonment and restructuring appeared to occur during this study both as a direct consequence of roost culling (i.e., Lihue_1, Lihue_3, and Koloa roosts) as well as for unknown reasons (i.e., Lihue_2; Fig. 1). Interestingly, three of the roosts that established during the study (Lihue_2, Lihue_3, and Koloa), were all previously documented roost sites prior to the study (Gaudioso et al. 2012; Shiels and Kalodimos 2019); therefore, it seems rose-ringed parakeets return to historic roost sites when contemporary roosts are disturbed. If managers in the future aim to identify movement of parakeets after roost abandonment, incorporating telemetry (Gaudioso et al. 2012) or aluminum neck collars with numbered tags (Senar et al. 2019) may be beneficial. Further, researchers may be able to identify potential roost sites by way of contemporary modeling approaches if a larger number of historical roost sites are documented.

The number of rose-ringed parakeets occupying individual roosts on Kaua‘i appears to be larger than others reported in the literature. Reported roost sizes in native habitats range from 498 to 1,111 individuals in Pakistan (Khan 2002) and 229 to 519 individuals in India (Shivaji et al. 2017). Roost size in invaded habitats appears to be more variable. In South Africa, four roosts were reported to include 20–100 individuals (Hart and Downs 2014). Maximum reported roost size in Portugal was 644 individuals (Luna et al. 2016). In Britain, roosts have been reported as small as four individuals (Pithon and Dynam 1999) and as large as 2,500 individuals (Butler 2003). The smaller documented sizes of roosts in other parts of the world may indicate roost culling would provide an impactful management strategy elsewhere.

Because this study was conducted during the COVID-19 pandemic, roost culling occurred with far fewer members of the public in view of culling activities than would have occurred during normal tourism and business operations. Public support can be critical to the success of environmental management programs (Jacobson 2009), and public opposition to invasive species management programs can lead to program termination (Bertolino and Genovesi 2003; Anderson et al. 2019). While public support of rose-ringed parakeet population control on Kaua‘i has not been rigorously surveyed, environmental outreach specialists on the island report that most residents of Kaua‘i are aware of the substantial impacts these birds are having and are supportive of lethal control efforts. Conversely, tourists to the island appear to be less supportive (T. Keanini, Kaua‘i Invasive Species Committee, *pers. comm.*). Thus, roost culling may be more challenging around tourist resorts on Kauaʻi (as is the case for the Poʻipū roost) outside of the COVID-19 pandemic. Similarly, it is possible there would be less public support for roost culling in other places where rose-ringed parakeets are invasive. Public support of invasive species’ lethal control programs is lower for birds and mammals than other taxa (Verbrugge et al. 2013). In Britain and Spain, management of nonnative parakeets was halted due to public response (Crowley et al. 2019; M. Sabaté, Departmento Téchnico, *pers. comm.*). Before implementing roost culling, managers should thoroughly consider public opinions and support and, if necessary, develop environmental outreach and education materials.

Understanding the biology and population dynamics of a target species is critical for population management. An effective management plan for invasive species population reduction requires an informed strategy including information on target numbers to be culled as well as the spatial distribution, duration, and timing of lethal removal (Grarock et al. 2014). It is unclear why the proportion of juvenile females removed in this study was higher than juvenile males. Little information exists on the influence of social structure or hierarchy on physical placement of rose-ringed parakeets within or among roosts. It is possible juvenile females are located in areas of the roost that are easier to target than other age or sex classes or that division among roosts is related to age and sex. It is also possible juvenile females represent a larger proportion in the population, perhaps due to higher survivorship, although to our knowledge this dynamic has also not been explored. Further, it is unknown whether the long-term shotgun culling on Kaua‘i has affected sex structure. With long-lived bird species, removing sexually mature individuals can have a greater impact on population suppression than removal of juveniles (Ellis and Elphick 2007). While many parrot species demonstrate monogamy (Spoon 2006), rose-ringed parakeets do not form life-long pair bonds; this may suggest culling adult females is more effective for reducing population growth than culling adult males. In this study, we found a larger proportion of sexually mature females among birds culled at the roost in May 2020 and February 2021 as compared to March and April 2020. This suggests future rose-ringed parakeet roost culling efforts on Kaua‘i should be restricted to months outside of March and April. Further research is merited to identify the breeding season of rose-ringed parakeets on Kaua‘i as well as at-nest metrics, such as clutch size and nest success rates. Population modeling could further evaluate the efficacy of culling each age and sex class.

Because roost culling only appears to be appropriate in some circumstances (e.g., where the need for culling outweighs the ethical considerations of potential sublethal injuries), managers should also consider alternative control options. Trapping was successfully used to capture and eradicate rose-ringed parakeets on the Canary Islands (Saavedra and Medina 2020) but was unsuccessful in the Seychelles (Bunbury et al. 2019). Prior to this study, researchers conducted a one-year trapping trial with a single trap on Kaua‘i that yielded no rose-ringed parakeet captures (Gaudioso et al. 2012). Mist netting success in capture of rose-ringed parakeets has varied in different locations (Butler 2003; Peck 2013; Bunbury et al. 2019; Mentil et al. 2019); to date, we are unaware of any efforts to capture rose-ringed parakeets via mist nets on Kaua‘i. Laboratory evaluations indicate the chemical contraceptive Diazacon effectively decreases rose-ringed parakeet fertility (Lambert et al. 2010); however, this has not yet been tested in field applications due to regulations and the need to avoid nontarget species. Culling via shotgun was effective when used along flight lines in the Seychelles (Bunbury et al. 2019). However, shotgun culling has been used for over 15 years in corn fields on Kaua‘i and population growth was not abated by these control efforts alone (Anderson et al. 2021). In reducing the rose-ringed parakeet population on Kaua‘i and other locations with widely-distributed nonnative populations, managers will likely need to coordinate multiple control methods and include adaptive management based on efficacy.

In addition to evaluating efficacy, managers must also evaluate potential impacts of control methods on native species. Because native wildlife species on Kaua‘i are largely restricted to the central uplands of the island, operators are able to conduct shotgun and roost culling in areas with very few native species present. In other introduced locations where native species occur in rose-ringed parakeet roosting or foraging sites, use of firearms may not be appropriate. Likewise, other control methods must be evaluated for potential impacts on native species. For example, chemical control should only be administered if non-target species are unable to access it, and traps must be designed to prohibit capture of non-target species.

As the rose-ringed parakeet range spreads and populations increase, managers will continually need informed management strategies to control populations or reduce populations to a level that increases efficacy of nonlethal tools to mitigate economic damages. While eradication has proven successful for small, incipient populations (Bunbury et al. 2019; Saavedra and Medina 2020), it may not be feasible for many invasive rose-ringed parakeet populations. Roost culling appears to be an efficient technique, but only appropriate for use in particular scenarios. Like other management tools, effective implementation of roost culling will need to be sustained, strategic, and implemented long-term. Culling should also be coupled with evaluations of population growth rate to determine efficacy. Future research of roost culling should more specifically evaluate the influence of culling frequency (e.g., culling on consecutive vs. non-consecutive nights at similar-size roosts), required shooter hours, as well as using alternative options to white spotlighting (e.g., infrared). Although further research is merited, it appears roost culling is a viable option to incorporate into integrated pest management programs for this invasive species.

## Supplementary Information

Below is the link to the electronic supplementary material.

Supplementary file1 (PDF 191 KB)

## Data Availability

The datasets generated during and/or analysed during the current study are available from the corresponding author on reasonable request.
