# Supplementary material for: Evaluation of roost culling as a management strategy for reducing invasive rose-ringed parakeet (Psittacula krameri) populations
Source: Biol Invasions. 2023 Jan 21;25(5):1403–19. doi: 10.1007/s10530-022-02984-3 (PMC9862218; doi:10.1007/s10530-022-02984-3)
Supplement: Supplementary file 1 — Supplementary file1 (PDF 191 KB) [file 10530_2022_2984_MOESM1_ESM.pdf]

**Supplementary Information**

# Evaluation of Roost Culling as a Management Strategy for Reducing Invasive Rose-Ringed Parakeet (*Psittacula krameri*) Populations

Biological Invasions

C. Jane Anderson[1], Leonard A. Brennan, William P. Bukoski, Steven C. Hess, Clayton D. Hilton, Aaron B. Shiels, Shane R. Siers, Bryan M. Kluever, Page E. Klug

[1]Corresponding Author. Caesar Kleberg Wildlife Research Institute, Texas A&M University–Kingsville. ecojaneanderson@gmail.com

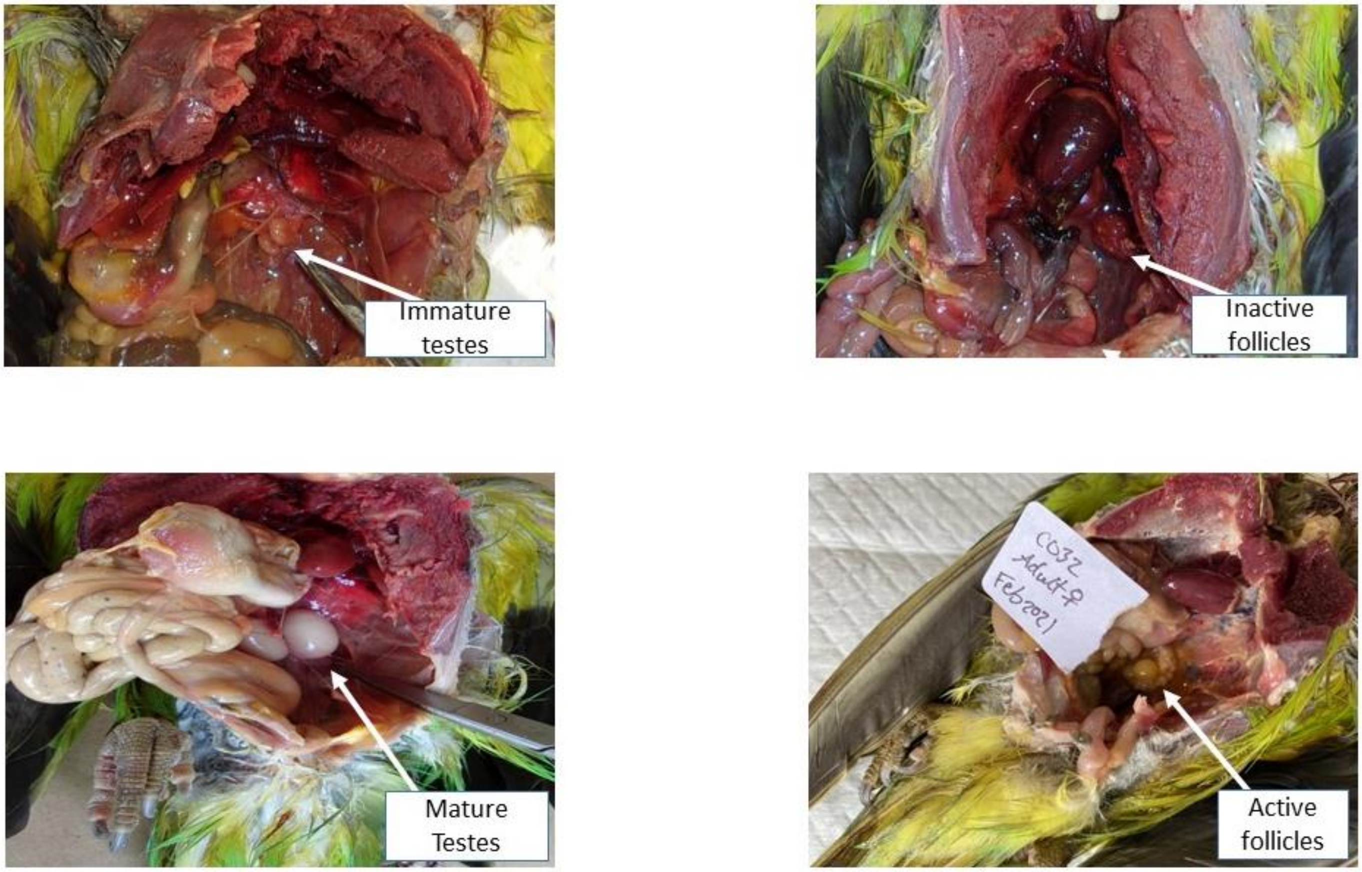


Figure S1. Gonads of rose-ringed parakeets culled on Kaua‘i, Hawai‘i, in 2020–2021. Upper left – sexually immature male. Upper right – sexually immature female. Lower left – sexually mature male. Lower right – sexually mature female.

```
###Multinomial Logistic Regression Code###

install.packages("nnet")
library(nnet)

###create dataframe###
Febdf = data.frame(AgeSex = c(rep("AF", length = 145), rep("AM", length = 229), rep("JF", length =
        47), rep("JM", length = 78)),
        Season = rep("Feb", length = 499)) #create dataframe of parakeets culled in February 2021
MrchAprldf = data.frame(AgeSex = c(rep("AF", length = 179), rep("AM", length = 415), rep("JF",
        length = 696), rep("JM", length = 221)),
        Season = rep("MrchAprl", length = 1511)) #create dataframe of parakeets culled in March and
        April 2020
Maydf = data.frame(AgeSex = c(rep("AF", length = 765), rep("AM", length = 627), rep("JF", length =
        1192), rep("JM", length = 832)),
         Season = rep("May", length = 3416)) #create dataframe of parakeets culled in May 2020

data = rbind(Febdf, MrchAprldf, Maydf) #combine three dataframes
data$Season = as.factor(data$Season) #ensure Season is factor
data$AgeSex = as.factor(data$AgeSex) #ensure AgeSex is factor

with(data, table(Season, AgeSex)) #verify values are correct

###run multinomial regression model###
data$AgeSex = relevel(data$AgeSex, ref="AF") #set reference level as adult female
model = multinom(AgeSex~Season, data = data) #run multinomial logistic model, with AgeSex as
dependent variable
summary(model) #look at model coefficients and standard errors

z = summary(model)$coefficients/summary(model)$standard.errors #calculate z scores
p = (1 - pnorm(abs(z), 0, 1)) * 2 #calculate p values
p
```
